# Influence of Social Media on Self-Medication Behavior: A Study on Ozempic Use Among Emirati University Students

**DOI:** 10.3390/ijerph23050638

**Published:** 2026-05-12

**Authors:** Riadh Jeljeli, Faycal Farhi, Faris El-Dahiyat, Mohammed Alsbou, Zaheer-Ud-Din Babar

**Affiliations:** 1 College of Arts, Humanities, and Social Sciences, University of Kalba, Sharjah 4444, United Arab Emirates; riadh.jeljeli@ukb.ac.ae; 2 College of Arts, Humanities, and Social Sciences, University of Al Dhaid, Sharjah 12600, United Arab Emirates; ffarhi@uodh.ac.ae; 3 Clinical Pharmacy Program, College of Pharmacy, Al Ain University, Al Ain 64141, United Arab Emirates; 4 Faculty of Medicine, Ajman University, Ajman P.O. Box 346, United Arab Emirates; 5 Department of Clinical Pharmacy and Practice, College of Pharmacy, Qatar University, Doha 2713, Qatar; z.babar@qu.edu.qa

**Keywords:** social media usage, Ozempic, semaglutide, weight loss, United Arab Emirates, Over Counter Medications (OTC)

## Abstract

**Highlights:**

**Public health relevance—How does this work relate to a public health issue?**

- This study sheds light on the rising issue of students using weight-loss medications without medical supervision, a behaviour increasingly influenced by social media.
- It draws attention to how online body image pressures are contributing to the non-prescribed use of Ozempic among university students in the UAE.

**Public health significance—Why is this work of significance to public health?**

- The findings show a clear link between heavy social media use, increased concern about body weight, and the likelihood of self-medicating for weight loss.
- By grounding the research in the Self-Medication Theory, the study helps explain how emotional distress and appearance-related pressures may push students toward unsafe health choices.

**Public health implications—What are the key implications or messages for practitioners, policy makers and/or researchers in public health?**

- There is a need for stronger awareness efforts within universities that promote healthy weight management and critical thinking about health information shared online.
- Health authorities and policymakers should reinforce regulations and educational campaigns to reduce the misuse of prescription medications for cosmetic weight loss.

**Abstract:**

Introduction: Self-medication has become a growing concern, especially in the current era of digitalization. The ubiquitous access to social media platforms has been associated with this behaviour due to factors like body image leading to weight loss obsession and seeking solutions to achieve the ideal body image and weight. Aims: This research also examined the relationship between Social Media Usage and Self-Medication, Weight Loss Obsession, and further Intention to Use Ozempic (semaglutide) among university students in the United Arab Emirates. Methods: The researchers used a cross-sectional design and gathered data from students enrolled at Al Ain University, United Arab Emirates. Data were analyzed using SPSS (Version 29) and SmartPLS software (Version 4). Descriptive statistics and Partial Least Squares Structural Equation Modelling (PLS-SEM) were employed to examine relationships among variables based on the Self-Medication Theory by Khantzian. Results: It was found that Social Media Usage was significantly linked with Self-Medication behaviour among the students. Also, this usage makes individuals conscious about weight and body image as important concerns. Finally, Social Media Usage was also significantly linked with Intention to Use Ozempic for weight loss without medical prescriptions and doctor consultations. Conclusions: It is concluded that self-medication for weight loss disregards the significance of maintaining a well-balanced diet and regular exercise, leading to serious health risks, i.e., nutritional deficiencies, metabolic disorders, and negative effects on mental well-being. Educating and informing young individuals about the importance of adopting healthy and sustainable weight loss processes is important, underlining the need for professional guidance, nutrition education, and promoting a positive body image. This approach may help reduce the harmful outcomes associated with self-medication for weight loss.

## 1. Introduction

Self-medication is a global concern mainly attributed to the use of drugs that may involve Over-the-Counter Medications (OTC), supplements, and conventional home remedies without the prescription or guidance of healthcare practitioners to treat self-identified healthcare issues [1]. Self-medication is not always a negative behaviour, showing interest in individuals towards their health and well-being [2]. On the positive side, self-medication reduces the loss of healthcare costs. However, it is a considerable phenomenon as it can lead to inappropriate, unsuitable utilization of drugs, delay in seeking medical care, and pathogenic resistance among individuals. According to Lukovic et al. [3], self-medication can also lead to misdiagnosis of disease and unsuitable consumption of drugs, further raising healthcare concerns. Keeping this phenomenon under consideration, one of the common reasons behind self-medication is the plethora of information and communication resources, such as social media, that significantly add more to one’s knowledge about the drugs, their availability, and usage. For instance, a recent study by Ali et al. [4] indicated social media platforms as one of the basic reasons behind raising healthcare challenges due to the availability of misinformation and drug-related information, further creating gigantic challenges for healthcare measures and practitioners. The availability of unfiltered informational resources, interpersonal communication patterns, and other resources remains prominent to challenge the current healthcare practices and public well-being. Carneiro et al. [5] argued that the attachment level to social media reflects the addictive nature of its usage, further leading to the availability of information, including drugs and their usage.

Similarly, self-medication among the younger generation is a concerning trend that has gained traction in recent years. With easy access to information and medical supplies, many individuals prefer to take self-medication into their own hands when treating various health conditions. In 2018, Gezer and Yalvaç [6] highlighted the weight loss obsession as an alarming phenomenon among youngsters, as many young people develop an unhealthy preoccupation with weight and body image in a society that stresses thinness and equates it with beauty and success. This obsession can lead to extreme dieting, excessive exercise, and the use of unregulated weight loss products and supplements. The consequences of this obsession can be severe, including malnutrition, eating disorders, and psychological distress. Moreover, pursuing an unrealistic body ideal can contribute to low self-esteem, body dysmorphia, and a distorted relationship with food [7]. As a result, international healthcare organizations recognize self-medication as a significant public health concern. While traditionally associated with the elderly population in the country, self-medication is widely observed among younger individuals [8].

Multiple studies have extensively reported the correlation between social media usage and risky behaviours, including anxiety, depressive symptoms, narcissism, and compromised sleep quality [9]. In addition, these studies also raised concerns about social media as a potential cause of Self-Medication behaviours among the young generation for weight loss purposes [5]. The present study examines three parallel outcome variables, Self-Medication behaviour, Weight Loss Obsession, and Intention to Use Ozempic (semaglutide), as independent dependent constructs influenced directly by social media usage. The study does not test a mediating structural pathway but rather assesses direct associations between variables within a PLS-SEM framework.

Based on the existing evidence, this research also assessed the role of social media usage among students in the United Arab Emirates in causing self-medication behaviours, particularly weight loss obsession. The primary gaps in the current literature can be identified by the fact that, despite many studies focused on the correlation between social media usage and weight loss obsession [10,11], they remained focused on certain geographical regions. As a result, this research focused on the United Arab Emirates to fill this gap. Second, this study employed the Self-Medication Theory as the theoretical underpinning. The relevant theory helped to fill the theoretical gap, as the relevant grounds are proposed for individuals with schizophrenia, alcoholism, and other relevant disorders [12]. Overall, this research would help future researchers, healthcare stakeholders, and general readers to investigate the perceived impacts of social media on self-medication behaviours among Emirati youth, which would further lead to proposing practical implications to ensure psychological healthcare and well-being of the young generation.

## 2. Literature Review

### 2.1. Ozempic (Semaglutide) for Weight Loss—Trends and Challenges

Semaglutide, marketed under the brand name Ozempic (semaglutide), is a glucagon-like peptide-1 receptor agonist used for weight management. One mL of the solution has 1.34 mg of semaglutide and is a human glucagon-like-peptide-1 (GLP-1) receptor agonist produced in Saccharomyces cerevisiae by recombinant Deoxyribonucleic acid (DNA) technology, followed by protein purification [13]. The drug has acquired attention recently due to its potential efficacy in assisting individuals with weight management. However, there are various trends and challenges associated with its use. One trend is the increasing popularity of Ozempic (semaglutide) as a weight loss solution, with more individuals striving for pharmaceutical assistance to reach their weight loss goals. According to Ojeniran et al. [14], the drug’s ability to suppress appetite and potentially improve metabolism makes it appealing to those toiling with obesity or weight-related health issues. This trend is further boosted by the influence of social media and online communities, where Ozempic (semaglutide) is often debated, revisited, and publicized.

On the other hand, the promotion of Ozempic (semaglutide) through social media platforms also raised many concerns. For instance, many individuals use Ozempic (semaglutide) without a doctor’s prescription. According to Singh et al. [15], Ozempic (semaglutide) usage requires prescription medication that should only be used under the supervision of a healthcare professional. Further, the potential side effects and interactions are also essential to consider, indicating self-medication and raising questions about its side effects and consumption patterns. Additionally, there are concerns regarding the long-term sustainability and usefulness of Ozempic (semaglutide) as a weight loss solution. The weight loss acquired through the drug may plateau over time, and discontinuing the medication can lead to weight regain. According to Wilding et al. [16], there may be individual divergences in response to Ozempic, with some individuals experiencing substantial weight loss while others may have minimal results.

### 2.2. The Self-Medication Theory

The basic conceptualization of current research is supported by the Self-Medication Theory [17,18], implying that people may engage in certain behaviours, i.e., substance abuse or addictive behaviours, to cope with underlying psychological or emotional issues. According to the Self-Medication Theory, people use these substances or demeanours as a form of self-medication to relieve symptoms of distress or psychological disorders. According to [19], Dr Ed Khantzian was important in bringing a more humane perspective to understanding addiction during the 1970s and 1980s. Together with other experts, Khantzian challenged the popular opinion that addiction stemmed from hedonism, sociopathy, or a desire for self-destruction. Instead, Dr Khantzian suggested that individuals struggling with alcoholism and addiction experience more intense and challenging emotional states than the general population. The theory of self-medication is based on the implications of psychological treatment in helping individuals in this cycle of self-medication and addiction [20].

Although the Self-Medication Theory was developed regarding psychological distress and substance use disorders, its application in the present study is conceptually extended to weight-related concerns. In this context, weight loss obsession is interpreted as a form of perceived psychological and emotional distress linked to body image dissatisfaction, which may lead individuals to engage in maladaptive coping behaviours such as self-medication [21,22]. This extension is consistent with empirical evidence showing that psychological distress and emotional dysregulation are closely related to maladaptive eating and weight-related behaviours, suggesting that emotional and cognitive distress can manifest in unhealthy coping strategies related to body weight and appearance [23]. However, the theory is applied within its conceptual boundary of explaining coping-driven behaviours instead of clinical psychopathology and is used to understand perceived distress and behavioural responses rather than diagnosed mental disorders.

Regarding the United Arab Emirates, recent data show that self-medication is common among residents from different age groups, sociocultural backgrounds, and ethnicities. Notably, community pharmacists in the UAE are allowed to access medicines without a doctor’s prescription. Specifically, they sell antibiotics and other relevant medicines due to common flu and fever, a lack of appropriate rules, and the absence of penalties for selling medicines without a prescription [24]. Thus, the conceptualization and underpinnings of the Self-Medication Theory can be assumed as suitable for current research, as this theory underlines social media usage for self-medication purposes [25], with the prior motive to lose weight by using Ozempic (semaglutide) as a popular medication. A growing body of research explores the connection between social media usage and self-medication behaviour. Social media platforms have become pervasive, providing individuals with constant information, relationships, and validation [26,27]. A study by Aksar et al. [10] also indicated the pervasive nature of social media, leading individuals to unrealistic beauty standards, comparison culture, and an emphasis on appearance, leading to body dissatisfaction, low self-esteem, and a desire to conform to societal expectations. Further, Carneiro et al. [5] also witnessed that negative emotions concerning body image and weight loss obsession, and achieving the desired body image, may lead some individuals to engage in self-medication behaviours, including extreme dieting, excessive exercise, or even resorting to unhealthy weight loss medications without a doctor’s prescription.

## 3. Materials and Methods

A cross-sectional survey was conducted at Al Ain University, United Arab Emirates. The relevant city and institutions were chosen because Al Ain is one of the most developed cities in the United Arab Emirates, having improved medical facilities, including private and public sector hospitals, pharmacies, and access to healthcare services. In addition, Al Ain University is one of the largest private sector universities in the region, with students from different regions worldwide. The data was collected over one month (1 May 2025 to 31 May 2025). The researchers emailed the survey questionnaires after having obtained their formal consent to participate.

### 3.1. Study Instrument and Measures

The data-gathering instrument was designed using items and scales from existing studies. The first section contained three questions regarding the demographics of study respondents, including their gender, age, and educational level. All constructs in this study were measured using a five-point Likert scale ranging from 1 (strongly disagree) to 5 (strongly agree). Higher scores indicate stronger agreement with the statements, reflecting higher levels of the respective construct. The Likert-scale approach allowed respondents to express the intensity of their agreement with each item used in the questionnaire. It is notable that the conceptual model treats all constructs as distinct endogenous variables influenced directly by social media usage, without specifying mediating relationships among them.

The construct validity of the modified scales was further reassessed. Convergent validity was evaluated using factor loadings and Average Variance Extracted (AVE), with all constructs exceeding the recommended threshold of 0.50. Discriminant validity was confirmed using the Fornell-Larcker criterion and HTMT ratios, all below the acceptable threshold of 0.85. These results indicate that the adapted scales retained adequate validity despite modifications.

Further, the questionnaire contained four sections, including questions about Social Media Usage, Social Media for Self-Medication, Weight Loss Obsession, and Intention to Use Ozempic (semaglutide) for Weight Loss Purposes (respectively). The following are the details about measurement items related to each construct.

i. Social Media Usage

The predictor “Social Media Usage” was measured by adopting items and scales from the study by [28]. In their study, Bohmer employed the relevant items to examine the use of social media among adolescents for different purposes. Current research adopted five items from the relevant research study and employed some formal formatting to match the study requirements. Table 1 shows the questionnaire items with a Cronbach’s alpha value of 0.791 (>0.7) and a composite reliability value of 0.865 (>0.7).

ii. Social Media Usage for Self-Medication

The second construct, “Social Media Usage for Self-Medication”, was adopted from the study by [25]. The relevant study used a descriptive design to examine social media for self-medication in the Philippines. However, the current study adopted six items from the study by Claire Mare-Armillo and their colleagues and employed them after adjusting them to the study’s topic, aims, and problems. Table 2 shows the questionnaire items with the Cronbach’s alpha value of 0.830 (>0.7) and a composite reliability value of 0.884 (>0.7).

iii. Weight Loss Obsession

The construct “Weight Loss Obsession” was measured by adopting measurement items from [29,30]. The current research adopted six items from both studies, as the first three items are from the study [29], and the remaining three are from [30]. These items were employed after making some necessary adjustments (language formatting and rephrasing). Table 3 shows the questionnaire items with a Cronbach’s alpha value of 0.755 (>0.7) and a composite reliability value of 0.855 (>0.7).

iv. Intention to Use Ozempic (semaglutide) for Weight Loss Purposes

The variable “Intention to Use Ozempic for Weight Loss Purposes” was measured by adopting questionnaire items from [31,32]. Notably, the items related to Ozempic were designed to measure a broader construct reflecting individuals’ perceptions, beliefs, and behavioural intentions toward its use for weight loss. Although these dimensions are conceptually related but distinct, they were combined to represent an overall orientation toward Ozempic as a weight-loss solution, consistent with earlier research combining attitudinal and intentional components. Accordingly, this construct is operationalized as an overall intention–attitude composite reflecting respondents’ cognitive beliefs and behavioural intentions toward semaglutide use for weight management.

However, both studies employed a seven-point Likert scale that was further adjusted and formatted for the current study and employed a five-point Likert scale. A total of eight items were selected from both studies (the first three from [31] and the remaining five from [32] for the data gathering). Table 4 shows the questionnaire items with a Cronbach’s alpha value of 0.855 (>0.7) and a composite reliability value of 0.897 (>0.7).

The structural model, therefore, evaluates direct relationships between Social Media Usage and each of the three outcome constructs independently.

### 3.2. Population and Sampling

The population of the current research was based on currently enrolled students from Al Ain University, Abu Dhabi. Recent data show 798 undergraduate students, 141 postgraduate/doctorate students, and 98 higher-level diplomas/certification students [33]. Thus, there are 1037 students in Al Ain University, Al Ain. This data calculation estimated the sample size using Krejci and Morgan’s formula [34]. The relevant formula indicated that a minimum sample size of 278 respondents would be ideal for the current study. The respondents were further randomly selected as there were no other inclusion/exclusion criteria, and survey questionnaires were distributed accordingly. The final response rate remained 89.2%, with 248 valid responses collected out of the targeted sample size of 278.

The gathered responses were further calculated once the data collection procedure was completed, indicating a response rate of 89.2%, which was higher than the minimum acceptable threshold of 60%. Based on the demographics of the respondents, among the 248 respondents, 81.0% were females, 18.5% were males, and 0.4% preferred not to disclose their gender. The age groups of the respondents were divided into four categories, where 48.0% were aged 30 years or above, 24.2% were between 21 and 24 years old, 21.0% were between 25 and 29 years old, and 6.9% were less than 20 years old. Finally, the educational level of the respondents indicated that 51.6% were undergraduate students, 28.6% were graduate-level students, 15.3% were postgraduate students, and 4.4% were doctorate-level students (refer to Table 5 for detailed descriptives).

### 3.3. Data Analysis

Descriptive measures were employed to examine the sample characteristics based on the investigated variables. Measures of central tendency were used to express descriptive data. Further, Partial Least Squares Structural Equation Modelling was conducted as an inferential analysis to test the relationship between predictor and dependent variables. Both SPSS (Version 29) and Smart-PLS (Version 4) were used for data analysis purposes.

### 3.4. Research Ethics

Every respondent received a thorough explanation regarding the confidential data handling before the survey commenced. A cover page seeking consent for participation was also provided to them. The respondent was assured that their data would be kept confidential. The participants also provided informed consent regarding their voluntary participation.

## 4. Analysis and Findings

### 4.1. Descriptive Analysis

The data analysis was based on two phases. First, the descriptives of the response were calculated. Later, structural equation modelling was executed. Table 6 indicates the results of descriptive analysis showing the means, standard deviation, range, and bootstrapping results of the gathered data. The results indicate that all variables are above the midpoint of the scale, suggesting generally high levels of Social Media Usage, Self-Medication behaviour, Weight Loss Obsession, and Intention to Use Ozempic (semaglutide) among respondents.

Among the variables, Intention to Use Ozempic (semaglutide) (M = 4.137) and Weight Loss Obsession (M = 4.1277) show the highest mean values, indicating relatively stronger agreement among participants regarding weight-related concerns and their willingness to use Ozempic (semaglutide). Social Media Usage (M = 4.0131) and Self-Medication (M = 3.7944) also indicate moderate to high levels of agreement.

The relatively low standard deviations across all constructs suggest that responses were fairly consistent among participants. In addition, the bootstrap confidence intervals indicate stability of the estimated means, as all intervals are narrow and do not cross low-scale values.

Further, Partial Least Squares Structural Equation Modelling (PLS-SEM) was conducted in two stages: (1) measurement model evaluation and (2) structural model evaluation.

### 4.2. Measurement Model Analysis

Internal consistency reliability was further confirmed using Cronbach’s alpha and composite reliability, with all constructs exceeding the recommended threshold of 0.70, indicating satisfactory reliability of the measurement model. Discriminant validity was assessed using the Fornell-Larcker criterion and the Heterotrait-Monotrait (HTMT) ratio. The results confirmed that each construct was empirically distinct, as the square root of AVE values was higher than the inter-construct correlations, and all HTMT values were below the threshold of 0.85. Thus, the measurement model demonstrated adequate reliability, convergent validity, and discriminant validity, confirming that the measurement scales were appropriate for further structural analysis.

**Figure 1:**
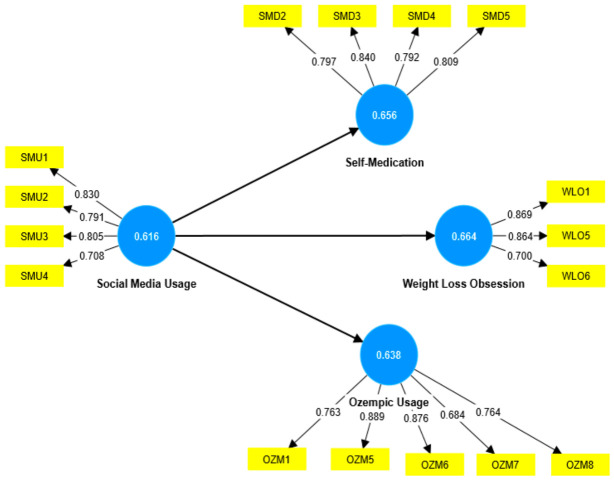
Measurement model results using PLS-SEM (SmartPLS). The figure presents factor loadings and construct relationships for Social Media Usage, Self-Medication, Weight Loss Obsession, and Intention to Use Ozempic (semaglutide), used to assess the reliability and validity of the measurement model.

The inner model analysis was first conducted to test the reliability and validity of the measurement model. First, the factor loads and Average Variance Extracted were calculated. Results showed that most of the factor loads surpassed the minimum threshold value of 0.5, while a few were less than the relevant value, which was further alleviated to ensure the goodness of fit. In addition, all the AVE values exceeded the minimum threshold value of 0.5 (Social Media Usage 0.616, Self-Medication 0.656, Weight Loss Obsession 0.664, and Ozempic (semaglutide) for Weight Loss Purposes 0.668). These results affirmed that all the constructs are internally consistent and reliable (refer to Figure 1).

The discriminant validity was assessed to ensure that the study constructs are empirically distinct. This was evaluated using the Fornell-Larcker criterion, based on inter-construct correlations within the study sample [35]. As shown in Table 7, the square root of AVE values for each construct was higher than the corresponding inter-construct correlations, confirming adequate discriminant validity.

Besides the second criterion, the “Heterotrait-Monotrait ratio (HTMT) Scale” was also applied. All the HTMT values were significantly less than the minimum threshold value of 0.85 (refer to Table 8). Thus, the results confirm satisfactory discriminant validity of the measurement model.

Before testing the structural model, the coefficient of determination (R^2^) values were assessed to examine the explanatory power of the model and the extent to which the independent variable explains variance in the dependent constructs. R^2^ is a key indicator in Partial Least Squares Structural Equation Modelling (PLS-SEM) as it reflects the predictive accuracy of the model. The results indicated that Social Media Usage explained 45.6% of the variance in Self-Medication behaviour (R^2^ = 0.456), 69.5% of the variance in Weight Loss Obsession (R^2^ = 0.695), and 56.7% of the variance in Intention to Use Ozempic (semaglutide) (R^2^ = 0.567). These results suggest that the model has moderate explanatory power for Self-Medication behaviour and Intention to Use Ozempic, and relatively strong explanatory power for Weight Loss Obsession.

Among the three endogenous variables, Weight Loss Obsession demonstrated the highest explained variance, indicating that Social Media Usage plays a particularly strong role in shaping attitudes and perceptions related to body image and weight concerns among respondents. This finding highlights the stronger psychological influence of social media on body-related cognition compared to behavioural outcomes such as Self-Medication. Furthermore, adjusted R^2^ values were also examined to account for model complexity and sample size, providing a more conservative estimate of explanatory power. The adjusted R^2^ values supported the robustness of the model, indicating that the observed explanatory power was not inflated by the number of predictors.

Effect size (f^2^) values were also assessed to evaluate the relative contribution of the predictor variable to each endogenous construct. The results indicated small to medium effect sizes, suggesting that Social Media Usage has a meaningful but varying level of influence across the dependent variables. All relationships were statistically significant at *p* < 0.001, as reported in Table 9, confirming the robustness and predictive relevance of the structural model. Overall, the findings demonstrate that the proposed model has acceptable explanatory and predictive capability within the study context.

### 4.3. Structural Model Analysis

Further, the structural model analysis was conducted using Partial Least Squares Structural Equation Modelling (PLS-SEM) to examine the hypothesized relationships between Social Media Usage, Self-Medication, Weight Loss Obsession, and Intention to Use Ozempic (semaglutide) [36]. The assessment of the structural model involved path coefficients, t-statistics, *p*-values, and confidence intervals to determine the strength and significance of the relationships. The findings revealed a statistically significant positive relationship between Social Media Usage and Self-Medication behaviour (β = 0.456, t = 11.513, *p* < 0.001). This indicates that higher levels of Social Media Usage are associated with increased tendencies toward Self-Medication among respondents. This finding is consistent with existing literature suggesting that social media acts as a key source of health-related information, which may influence medication-related decisions without professional consultation.

Similarly, a strong positive relationship was found between Social Media Usage and Weight Loss Obsession (β = 0.695, t = 10.424, *p* < 0.001). This represents the strongest relationship observed in the model, indicating that Social Media Usage is highly associated with increased body image concerns and weight-related preoccupation among university students. This suggests that exposure to idealized body standards on social media platforms may significantly shape individuals’ perceptions of body image and weight management behaviours. In addition, a significant positive relationship was identified between Social Media Usage and Intention to Use Ozempic (semaglutide) for weight loss purposes (β = 0.567, t = 7.846, *p* < 0.001). This suggests that individuals with higher exposure to social media content are more likely to report intentions to use pharmacological weight loss interventions. The result highlights the potential influence of online information and digital platforms in shaping attitudes toward medical weight loss solutions. It is also important to note that although Ozempic (semaglutide) has been clinically evaluated for weight management, its observed association in this study reflects respondents’ perceptions and intentions rather than confirmed clinical usage [16] (P.993). This distinction is important given the cross-sectional nature of the study.

Therefore, all hypothesized relationships were found to be statistically significant at *p* < 0.001, confirming that Social Media Usage plays a meaningful role in shaping Self-Medication behaviour, Weight Loss Obsession, and Intention to Use Ozempic (semaglutide). The results are further supported by confidence intervals and total effect estimates, indicating robustness of the structural model (Table 10).

## 5. Discussion on Results

Self-medication is a growing concern in the United Arab Emirates (UAE). This trend is associated with various factors, including increasing healthcare costs, busy lifestyles, and little awareness about the risks associated with self-diagnosis and treatment. Several studies witness self-medication as an increased concern in the Middle Eastern Region [37,38,39]. A study by Abasaeed et al. [36] also witnessed that 46% of individuals in the United Arab Emirates prefer self-medication without a formal medical consultation. In addition, 28% reported storing home medicines acquired from community pharmacies without a prescription. Talking specifically about the young generation, a study by Sharif et al. [40] indicated that 86% of university students prefer self-medication, mainly antibiotics and painkillers, for miscellaneous purposes. However, the relevant study also indicated that self-medication is mainly attributed to non-serious health problems concerning minor health issues, avoiding spending long hours in clinics, and seeking instant relief [41].

The current research also witnessed self-medication for weight loss among the young generation as a concerning trend due to social media usage and the availability of several information resources. Many young individuals, driven by societal pressure and unrealistic beauty standards, resort to self-medication through unregulated diet pills, supplements, or extreme dietary constraints without appropriate medical supervision [5]. Consequently, several medications ensure weight loss and better healthcare without medical prescriptions and formal consultation. Study respondents revealed that they take drugs/remedies for self-medication that they read on social media sites, frequently browse social media to seek information related to medicines, and regard the available information without a doctor’s prescription. In addition, the respondents consider the relevant information to pose no risk regarding self-medication, which further helps them manage their health condition. Moreover, the respondents also acquire medicines easily from community pharmacies. These results are consistent with the study by Claire Marie-Armillo et al. [25], as they witnessed social media for self-medication among young university students in Manilla, Philippines. The study respondents also agreed that they consider social media to be accelerating fitness trends.

Consequently, they are considering diet-taking habits and preferring thin bodies as part of a healthy lifestyle. The respondents also agreed that they feel the urge to lose weight after watching individuals on social media and comparing their bodies with those of others. As a result, they also feel pressure to lose weight by using available medications. These results remained in line with the studies by Nasir [29] and Moty et al. [30], indicating the impact of social media on body image and increased eating disorders among the users in Pakistan and Mauritius (accordingly). Finally, the study respondents also strongly agreed with social media usage and Ozempic (semaglutide) for weight loss purposes. According to them, they believe in the importance of medicines for weight loss purposes and prefer using anti-obesity drugs available online. The respondents also expressed that they know the importance of Ozempic (semaglutide) for weight loss as an effective medicine. In addition, the respondents also believe that the online information about Ozempic (semaglutide) is reliable and can be taken without a doctor’s suggestion/prescription. The respondents also agreed that they could take Ozempic (semaglutide) to lose weight in the future, and the available information about it is reliable. However, it is crucial to report that these findings reflect respondents’ perceptions and intentions toward Ozempic (semaglutide) use instead of confirmed actual consumption behaviour. These results indicate consistency with the argument by Burki [42], witnessing the role of social media, particularly TikTok, in surging the demand for Ozempic. Furthermore, Burki raised concerns about the increased demand for Ozempic for weight loss among users and indicated a shortage of Ozempic (semaglutide) for individuals with Type 2 Diabetes that will likely continue during the upcoming years.

### 5.1. Policy Implications

The findings of this study have some important policy implications for public health authorities, universities, and digital health stakeholders in the United Arab Emirates. A key implication is that Weight Loss Obsession plays a critical role in linking Social Media Usage with both Self-Medication behaviour and Intention to Use Ozempic (semaglutide). This suggests that policy responses should go beyond general health promotion and directly address psychological and social drivers of weight-related behaviours. In particular, interventions should focus on reducing body image anxiety and appearance-based social comparison triggered by social media exposure. Public health campaigns should target unrealistic beauty standards commonly promoted on digital platforms and raise awareness about the psychological impact of such content on young individuals.

Furthermore, digital health literacy programmes should be strengthened within universities to help students critically evaluate health and weight-loss information obtained from social media. These programmes should emphasize the risks of self-medication and the importance of professional medical consultation before using pharmacological weight-loss interventions. Finally, collaboration between healthcare authorities and social media platforms may also be beneficial in regulating misleading or unverified content related to weight loss medications, including Ozempic (semaglutide). Hence, targeted psychological and digital interventions are more likely to be effective than general lifestyle recommendations in addressing the behavioural outcomes identified in this study.

### 5.2. Conclusions

This research contributes as a baseline study to indicate a potential relationship between Social Media Usage, Self-Medication, Weight Loss Obsession, and Ozempic (semaglutide) for weight loss purposes in the United Arab Emirates. Although self-medication for other purposes is increasing, the parameters of an ideal body image and their attribution to healthcare have influenced interest in finding new ways to fulfil these parameters. The wider availability of social media and information resources has also contributed much to shaping perceptions of ideal body image, leading to a greater inclination toward accessing and using drugs like Ozempic (semaglutide) for weight loss. Previous studies have also witnessed the relevant phenomenon and raised concerns about the plethora of information available through these social media platforms. This research concludes that self-medication for weight loss is associated with reduced emphasis on a balanced diet and regular exercise, posing serious health risks, i.e., nutritional deficiencies, metabolic disorders, and adverse effects on mental well-being. Appraising young people about the importance of healthy and sustainable weight loss techniques is critical, emphasizing the significance of professional guidance, nutrition education, and encouraging positive body image to help reduce the harmful outcomes associated with self-medication for weight loss.

### 5.3. Limitations and Future Research

This study has some common limitations based on the scope and generalizability. First, this study has a limited scope as it only examined the students currently enrolled at Al Ain University. Future researchers can delimit by conducting similar studies in other institutions to gain further insights. Second, it is also acknowledged that the sample was drawn from a single university and may not be fully representative of the broader student population, which limits the generalizability of the findings. Future studies can address this limitation by collecting data from multiple universities and a more diverse student population to improve the generalizability and robustness of the findings. This further study involved Ozempic (semaglutide) as attributed to social media and self-medication, while there are many other medicines, such as dulaglutide (Trulicity), liraglutide (Victoza), and others. Future studies can focus on other medications for weight loss purposes and provide strong insights into the role of social media in affecting self-medication, body image, and weight loss medication usage among individuals. Finally, the sample was not equally distributed in terms of gender, which may further affect the representativeness of the results across different demographic groups. Future studies can address these limitations by collecting data from multiple universities and ensuring a more balanced and diverse sample to improve the generalizability and robustness of the findings.

## Figures and Tables

**Table 1:** Measurement items for Social Media Usage.

#	Scale Items	Source(s)
1.	For me, social media usage is important to avail health-related information.	[28]
2.	For me, social media usage is important to attain information about healthcare-related events.	
3.	For me, social media usage is important to avail healthcare advice.	
4.	For me, social media usage is important for reading interesting information about healthcare and well-being.	
5.	Social media usage is important for me as I can judge my and others’ physical appearance.	

**Table 2:** Measurement items for Social Media Usage and Self-Medication.

#	Scale Items	Source(s)
1.	I take drugs/remedies for self-medication that I read on websites.	[25]
2.	I frequently browse social media to find information about medicines.	
3.	I frequently take information about medications from social media without a doctor’s suggestion/prescription.	
4.	I consider information on social media poses no risk regarding self-medication.	
5.	I consider the available information to help me to manage my healthcare condition.	
6.	I purchase medicines from the community pharmacy easily.	

**Table 3:** Measurement items for Social Media Usage and Weight Loss Obsession.

#	Scale Items	Source(s)
1.	I consider social media to accelerate fitness trends among users.	[29,30]
2.	I consider social media as affecting my diet-taking habits.	
3.	I consider social media to encourage thin bodies as a part of a healthy lifestyle.	
4.	I feel the urge to lose weight after watching individuals on social media.	
5.	I compare my body with those who appear on social media.	
6.	I feel pressure to lose weight by using medications available on the market.	

**Table 4:** Measurement items for Intention to Use Ozempic (semaglutide) and Weight Loss Obsession.

#	Scale Items	Source(s)
1.	I believe in the importance of medicines for weight loss purposes.	[31,32]
2.	I prefer using anti-obesity drugs available online.	
3.	I am aware of the use of Ozempic (semaglutide) for losing weight.	
4.	I believe Ozempic(semaglutide) is effective in losing weight.	
5.	I believe online information about Ozempic (semaglutide) is reliable.	
6.	I believe Ozempic (semaglutide) can be taken without a doctor’s suggestion/prescription.	
7.	I believe I can take Ozempic (semaglutide)to lose weight in the future.	
8.	I believe online information about Ozempic (semaglutide).	

**Table 5:** Calculations of respondents’ demographics.

Constructs	Categorization	*N*	%	Bootstrap 95% Confidence Interval	
				Lower	Upper
Gender	Male	46	18.5	14.1	23.8
	Female	201	81.0	75.8	85.5
	Prefer Not to Say	1	0.4	0.0	1.2
Age	Less than 20 years	17	6.9	4.0	10.1
	21–24 years	60	24.2	19.0	29.4
	25–29 years	52	21.0	15.7	25.8
	30 years or above	119	48.0	42.3	54.0
Educational Level	Undergraduate	128	51.6	45.2	57.3
	Graduate	71	28.6	23.4	33.9
	Postgraduate	38	15.3	11.3	19.8
	Doctorate	11	4.4	2.0	7.3

**Table 6:** Descriptive of gathered data.

Variable	*M*	*SD*	Range	Bootstrap	
				95% Confidence Interval	
				Lower	Upper
Social Media Usage	4.0131	0.70105	3.25	3.9254	4.1048
Self-Medication	3.7944	0.80739	4.00	3.6946	3.8942
Weight Loss Obsession	4.1277	0.76254	3.33	4.0256	4.2285
Intention to Use Ozempic (semaglutide)	4.137	0.82812	3.40	4.0259	4.2387

**Table 7:** Fornell-Larcker criterion.

	Social Media Usage	Self-Medication	Weight Loss Obsession	Intention to Use Ozempic (Semaglutide)
Social Media Usage	0.799			
Self-Medication	0.121	0.810		
Weight Loss Obsession	0.47	0.379	0.785	
Intention to Use Ozempic (semaglutide)	0.462	0.501	0.56	0.815

**Table 8:** Heterotrait-Monotrait ratio (HTMT) Scale.

Constructs	Heterotrait-Monotrait Ratio (HTMT)
Self-Medication ↔ Intention to Use Ozempic (semaglutide)	0.199
Social Media Usage ↔ Intention to Use Ozempic (semaglutide)	0.562
Social Media Usage ↔ Self-Medication	0.439
Weight Loss Obsession ↔ Intention to Use Ozempic (semaglutide)	0.56
Weight Loss Obsession ↔ Self-Medication	0.63
Weight Loss Obsession ↔ Social Media Usage	0.687

**Table 9:** Coefficients of determination R square.

Strength	*R* ^2^	Sign
Social Media Usage → Self-Medication	0.456	0.000
Social Media Usage → Weight Loss Obsession	0.695	0.000
Social Media Usage → Intention to Use Ozempic (semaglutide)	0.567	0.000

*R* Square Adjusted = 0.205, *f*^2^ = 0.167, Sign = 0.000. *R* Square Adjusted = 0.481, *f*^2^ = 0.456, Sign = 0.000. *R* Square Adjusted = 0.319, *f*^2^ = 0.283, Sign = 0.000.

**Table 10:** Path coefficients, regression weights.

	Path Coefficients	STDE	*t* Statistics	Confidence Intervals		Sign
				2.5%	97.5%	
Social Media Usage → Self-Medication	0.456	0.053	11.513	0.307	0.621	0.000
Social Media Usage → Weight Loss Obsession	0.695	0.054	10.424	0.564	0.824	0.000
Social Media Usage → Intention to Use Ozempic (semaglutide)	0.567	0.061	7.846	0.419	0.704	0.000

Total Effect: 0.379, 0.560, 0.470 (Respectively).

## Data Availability

The data presented in this study are available from the corresponding authors upon reasonable request.
